# Metallic Wood through Deep-Cell-Wall Metallization: Synthesis and Applications

**DOI:** 10.1021/acsami.4c02779

**Published:** 2024-04-18

**Authors:** Xiaoying Xu, Jonas Garemark, Farsa Ram, Zhen Wang, Yuanyuan Li

**Affiliations:** † Wallenberg Wood Science Center, Department of Fiber and Polymer Technology, KTH Royal Institute of Technology, SE-10044 Stockholm, Sweden

**Keywords:** metallic wood, cell-wall nanoengineering, wood metallization, diffusion, multilayered cell wall, stress sensor

## Abstract

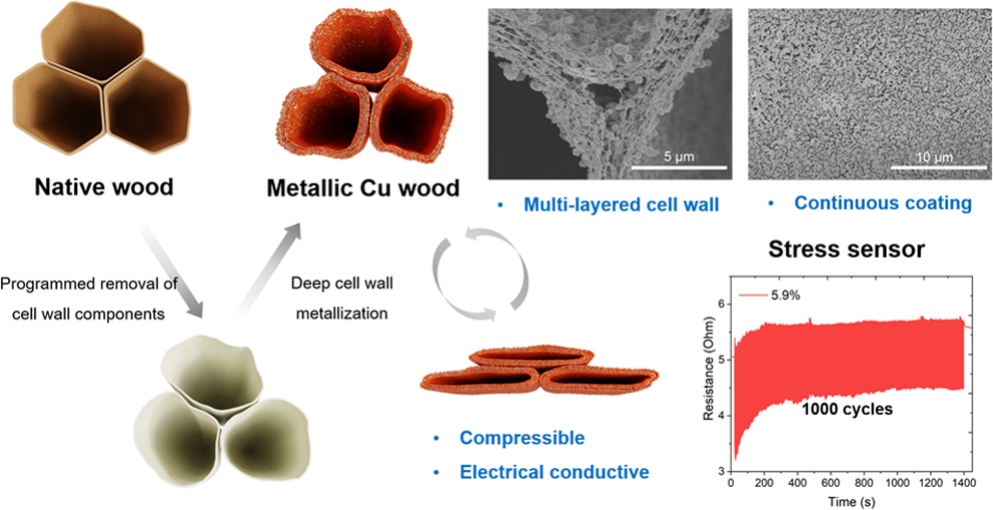

Metallic wood combines the unique structural benefits of wood and the properties of metals and is thus promising for applications ranging from heat transfer to electromagnetic shielding to energy conversion. However, achieving metallic wood with full use of wood structural benefits such as anisotropy and multiscale porosity is challenging. A key reason is the limited mass transfer in bulk wood where fibers have closed ends. In this work, programmed removal of cell-wall components (delignification and hemicellulose extraction) was introduced to improve the accessibility of cell walls and mass diffusion in wood. Subsequent low-temperature electroless Cu plating resulted in a uniform continuous Cu coating on the cell wall, and, furthermore, Cu nanoparticles (NPs) insertion into the wood cell wall. A novel Cu NPs-embedded multilayered cell-wall structure was created. The unique structure benefits compressible metal-composite foam, appealing for stress sensors, where the multilayered cell wall contributes to the compressibility and stability. The technology developed for wood metallization here could be transferred to other functionalizations aimed at reaching fine structure in bulk wood.

## Introduction

Metals are well-known for their excellent electrical conductivity, reactivity, and/or catalytic properties, thus being intensively explored in batteries, supercapacitors, power generators, etc.^1,2^ The rational structure design of metals, e.g., directional pores, could progress beyond their current performance and applications. Natural materials have an impressive, sophisticated configuration, which is inspiring for metal structuring. For example, wood is an assembly of aligned cells with anisotropic and hierarchical structure from molecular to meter scale. The organized hollow fibers with micro- and nanosized channels are able to transport water, ions, and minerals for the growth of trees. The oriented cellulose fibrils in the matrix of hemicellulose and lignin provide mechanical support.^3^ In addition, renewable sources, low cost, large-scale processability, and biodegradability of wood offer additional benefits. Therefore, metal structuring via biomimicking wood is attractive for obtaining composites with combined wood structural benefits and metallic properties, termed metallic wood here.

The introduction of metals into wood templates has been conducted for decades. Nickel (Ni)-plated wood veneers were reported for electromagnetic shielding, where a thin film of Ni was coated on wood substrates, appealing for replacing conventional shielding materials such as bulk metal or alloy.^4,5^ Lithium (Li)-infiltrated wood was explored as room-temperature Li-ion batteries.^6^ The well-aligned wood cells prevented the formation of Li dendrite by avoiding volume change during Li stripping/plating, enabling long-term cycling stability. The aligned pore arrays could also overcome the restricted diffusion of Li ions caused by tortuous porosity in thick electrodes.^7^ In addition, the waveguide effect resulting from the multiscale porosity of wood and the plasmonic performance of metal nanoparticles (NPs; palladium, gold, and silver) gave the metal-deposited wood great light absorption ability, thereby making it promising for solar steam generation.^8^ Metallic wood was also used for anisotropic conductivity^9^ and as a corrosion-resistant composite.^10^

Metallization of wood is generally performed with direct-current (DC) sputtering, melt diffusion, and electroless plating. DC sputtering is a thin film deposition technique that utilizes ionized gas to sputter molecules off the target material into plasma and deposit them onto the wood substrate.^11^ The surface being the only reachable part is an issue of physical vapor deposition. Melt infusion could work for the metallization of bulk materials by filling the wood template with molten metals or alloy via capillary action. For example, stannum–bismuth (Sn–Bi) alloy was filled into the pretreated pine for electromagnetic shielding,^9^ and molten Li^6^ or sodium (Na)^12^ was rapidly infused in carbonized wood to prepare high-performance anodes. However, melt infusion is limited to metals of low melting point. The original porosity of wood templates was compromised as well. Electroless plating, also named chemical/autocatalytic plating, is promising to access the internal structure and preserve the structural advantages of wood. In electroless plating, the substrate is placed in a plating bath (including metal cations, buffer, complexing agent, reducing agent, and stabilizer),^12^ where autocatalytic reduction of metal cations and deposition take place. Electroless Ni plating of wood veneers has been reported for electromagnetic shielding materials since 2006.^4,5,13^ The thickness of wood veneers was usually less than 1 mm, and plating generally occurred on the surface. Copper (Cu) plating of wood was also investigated with the purpose of superhydrophobicity^14^ and antibacterial properties,^15^ at which Cu(II) was reduced by (dimethylamino)borane (DMAB) and attached to the wood surface through the chelating ability of polydopamine. In situ carbonization was reported to synthesize Cu–wood composites as well.^16,17^ During the process, Cu precursor was reduced to metallic Cu under high temperatures (generally >500 °C), while biopolymers in wood were transferred into carbon. The energy-intensive process is a challenge to scalable production and the potential for modifications was reduced due to the chemical inertia of carbon materials.

Electroless metal plating of wood, termed wood metallization here, on its fine structure is challenging mainly due to limited mass diffusion and the vigorous rate of metal-ion reduction. Take balsa wood (*Ochroma pyramidale*) as an example; around 70% of cells are fibers,^3^ which have closed ends and lengths of 0.2–1.2 mm. Mass transfer along the fibers is restricted in thick samples. Vessels (wide cells with diameters of around 200–350 μm and wholly or partly open ends) help chemical diffusion, but they only account for 3–9 vol %.^18^ Pits are thinner portions of the cell wall responsible for communication and fluid exchange between adjacent cells.^18^ However, in the case of metal deposition, pits would be easily blocked by newly formed NPs, making the diffusion of chemicals into bulk structure even harder. Wang et al. regulated the reaction kinetics of electroless Cu plating by decreasing the temperature, which enabled metallization throughout the porous cellulosic Whatman chromatography paper.^19^ The microstructure of the starting materials is well preserved, and fibers are continuously coated with Cu claddings. In spite of its success in porous cellulosic paper, wood metallization via electroless plating is generally restricted to wood or cell-wall surfaces. To the best of our knowledge, deep metallization of thick wood templates, in other words, metallization on a fine structure such as an inner cell wall, has not been achieved.

**Figure 1:**
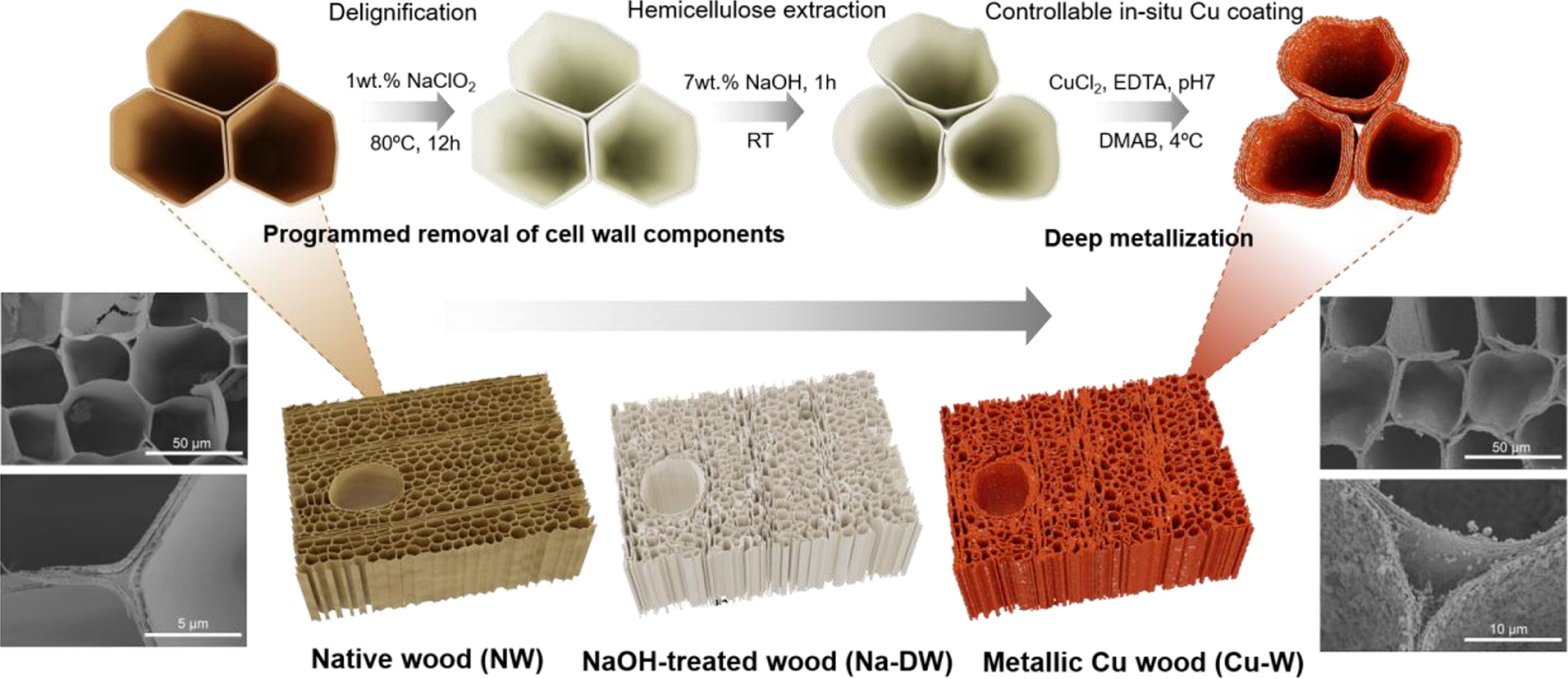
Schematic illustration of Cu-W preparation. Delignification, hemicellulose extraction, and electroless Cu plating, as well as the corresponding cell-wall structure variation, are shown. Schematics at the bottom depict the anisotropic structure of wood templates. SEM images on the left show the original structure of NW, while the final Cu-W (SEM image on the right) displays continuous Cu coating on the cell wall and a multilayered cell wall embedded with Cu NPs.

Controlled removal of cell-wall constituents could result in wood with higher porosity and better accessibility, favorable for deep wood metallization. Delignification is usually applied to increase wood accessibility yet with limited improvement.^20^ Hemicellulose removal after delignification was also investigated. However, the reported hemicellulose removal was generally performed under high temperature (80–100 °C) for a long time (5–8 h),^21,22^ leading to severe damage to the wood cell structure. In this work, controlled removal of cell-wall components was proposed to significantly improve accessibility and chemical diffusion while preserving the cell-wall structure. Specifically, delignification followed by room-temperature NaOH treatment was introduced in order to generate a high cell-wall porosity. Cu is selected as the target metal due to its high electrical conductivity, abundance, and recyclability.^23^ Low-temperature electroless Cu plating was then applied to slow the kinetics of Cu(II) reduction and maximize diffusion of the reducing agent. In this case, blockage of the openings due to rapid NP formation will be prevented, thus facilitating metallization to the inner structure of wood. Figure 1 illustrates the procedure of deep wood metallization in the cell wall. Continuous Cu coating with NP clusters was well plated on each cell wall, and a novel multilayered cell-wall structure embedded with Cu NPs was created as well. The structure merits of wood, such as anisotropy and multiscale porosity, are preserved. Additionally, metallic wood demonstrated compressibility and electrical conductivity, making it promising as a stress sensor.

## Experimental Section

### Materials and Chemicals

Balsa wood (*O. pyramidale*) of density 102 ± 5 kg/m^3^ was bought from Material AB, Sweden. Samples of dimensions 15 × 15 × 5 mm^3^ (tangential × radial × axial) were cut for chemical treatment. Sodium chlorite (NaClO_2_, 80%), sodium hydroxide (NaOH, ≥98%), copper chloride dihydrate (CuCl_2_·2H_2_O, ≥99.0%), ethylenediaminetetraacetic acid (EDTA, [(CH_2_COOH)_2_NCH_2_]_2_, 99.4–100.6%), boric acid (H_3_BO_3_, ≥99.5%), and (dimethylamino)borane (DMAB, (CH_3_)_2_NHBH_3_, ≥97%) were bought from Sigma-Aldrich, Sweden, and ethanol (96%) was bought from VWR, Sweden.

### Delignification of Balsa Wood

Balsa wood was delignified in 1 wt % NaClO_2_ in an acetate buffer (pH 4.6) solution at 80 °C for 12 h.^24^ The solution was renewed every 6 h. Wood templates were then repeatedly washed in deionized water until chemicals were removed.

### NaOH Treatment

Wood templates were introduced to a 7 wt % NaOH solution. Wood soaked in NaOH was kept at room temperature for 10 min to 2 h, and a vacuum was used to help diffusion of NaOH. Subsequently, NaOH was washed off from wood templates.

### Wood Metallization

The electroless plating bath included 0.25 M CuCl_2_, 0.25 M EDTA, and 0.375 M H_3_BO_3_, and the solution was neutralized to pH 7 using NaOH. Borate buffer kept the pH from changing too fast and stabilized the growth of NPs. Complexing agent (EDTA) prevented the precipitation of metal-ion salts and reduced the concentration of free ions.^12^ With the help of a buffer and a complexing agent, the Cu plating was more stable and well controlled. Samples were soaked in a CuCl_2_–EDTA complex overnight to ensure that the templates were fully infiltrated by the Cu plating bath. DMAB (0.375 M) was dissolved in CuCl_2_–EDTA in an ice bath, and CuCl_2_–EDTA-soaked samples were then introduced to the system. The plating process was carried out in the fridge at 4 °C to decrease the reduction rate of NPs and maximize diffusion of the reducing agent into the wood template. The electrochemical reactions of Cu plating^25^ are tabulated in Table S1. During this time, 5 min of degassing was run every day to remove gaseous products and promote the plating reactions. When the complex solution turned from sapphire blue to a clear colorless liquid, indicating that Cu plating was done, which usually takes 7 days, ethanol was used to stop the plating reactions and the generation of copper oxide. Afterward, metallic Cu wood (Cu-W) was washed with deionized water to remove the buffer and then freeze-dried for further characterizations.

### Characterization

The morphology was observed by field-emission scanning electron microscopy (SEM; Hitachi S-4800, Japan). Cross sections of wood were prepared by freeze-fracture in liquid nitrogen (−196 °C), followed by freeze-drying (−110 °C) for at least 48 h. All samples were sputter-coated prior to analysis with a gold/palladium coating of ≈5 nm, using a Cressington 208HR sputter coater (U.K.) for 25–30 s. Energy-dispersive X-ray spectroscopy (EDX; Oxford Instruments X-MAX N 80, U.K.) was used for elemental analysis, where the accelerating voltage was 20 kV to stimulate the characteristic X-rays of Cu. Mapping mode was chosen to evaluate the presence and distribution of elements.

The specific surface area (SSA) was obtained by nitrogen physisorption using 3Flex Micromeritics. Before measurement, samples were degassed at 90 °C for 3000 min to remove the absorbed contamination and access to all surface area in samples. Samples were analyzed in the relative pressure (*P*/*P*_0_) range of 0.05–0.995 in liquid nitrogen (−196 °C). The SSA was determined in the relative pressure range of 0.05–0.25 via the Brunauer–Emmett–Teller (BET)^26^ model.

Functional groups were characterized using a PerkinElmer Spectrum 100 Fourier transform infrared (FTIR) spectrometer. The spectra were obtained over the range of 4000–600 cm^–1^.

X-ray diffraction (XRD) was performed using a PANalytical X’Pert Pro powder diffractometer through Cu Kα radiation at 40 mA and 45 kV. The scans were performed over 2θ of 10–80° with a step size of 0.01° and a scan speed of 0.02°/s. To evaluate the contributions from crystalline and amorphous components, Gaussian deconvolution was used for curve fitting.^27^ The diffraction spectra were deconvoluted using Gaussian profiles in *OriginPro*, and iterations were performed until the coefficient of determination *R*^2^ reaches 0.996 in all deconvolution cases. Figure S1 shows the diffraction pattern of native wood (NW), fit peaks at various band positions since deconvolution, and cumulative fit peak. The crystallinity index (CI) could be calculated from the ratio of the area of all crystalline peaks to the total area^28^ (eq 1).
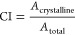
1

The lignin and carbohydrate contents were obtained by grinding the samples using a Wiley mill, followed by hydrolyzing the crushed materials in 72% sulfuric acid at room temperature and then 2.48% sulfuric acid in an autoclave at 125 °C for 1 h. The hydrolyzed substance was thereafter filtered to separate the lignin from the carbohydrates. The lignin content was subsequently obtained through the standard method: TAPPI T 222 om-2. The filtered solution was diluted and introduced to a Dionex ICS-300 ion chromatograph (Thermo Fisher Scientific Inc.) for analysis of the carbohydrate constituents.

The porosity (*f*) of the specimen was calculated by using eq 2. The bulk densities were obtained by drying the samples at 105 °C overnight or freeze-drying, followed by measuring the dimensions with a caliper and weighing the dry specimens. The solid density of balsa wood (cellulose) was assigned as 1500 kg/m^3^.^29^
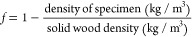
2

For the diffusion test, samples with dimensions of 10 × 3 × 500 mm^3^ (tangential × radial × axial) were freeze-dried and fixed at the same height. Nonfixed ends of the samples were dipped in a CuCl_2_–EDTA solution at the same time. A video was recorded, and the moving distance of the blue solution was measured by *ImageJ*, at which time eight values were averaged.

The mechanical properties were evaluated through tangential compression in an Instron 5944, utilizing a 10 kN load cell at a strain rate of 50 mm/min, 23 °C, and 50% relative humidity. All specimens were cut in half with dimensions of 7.5 × 15 × 5 mm^3^ (tangential × radial × axial) to avoid incidental bending during compression.

The stress sensor was assembled by gluing the metallic wood on Cu tape, Cu-plated wood was glued on Cu tape, leaving the side corner open to avoid a short circuit. Cu wires were soldered on the Cu tape to ensure the connection for the measurements. The electrical resistance was recorded by a Keithley DMM 7510. The device was mounted on a stage equipped with a linear motor system (Linmot, USA) to apply a periodic force on the device. Through control of the end position, different strains could be exerted on the test sample.

## Results and Discussion

**Figure 2:**
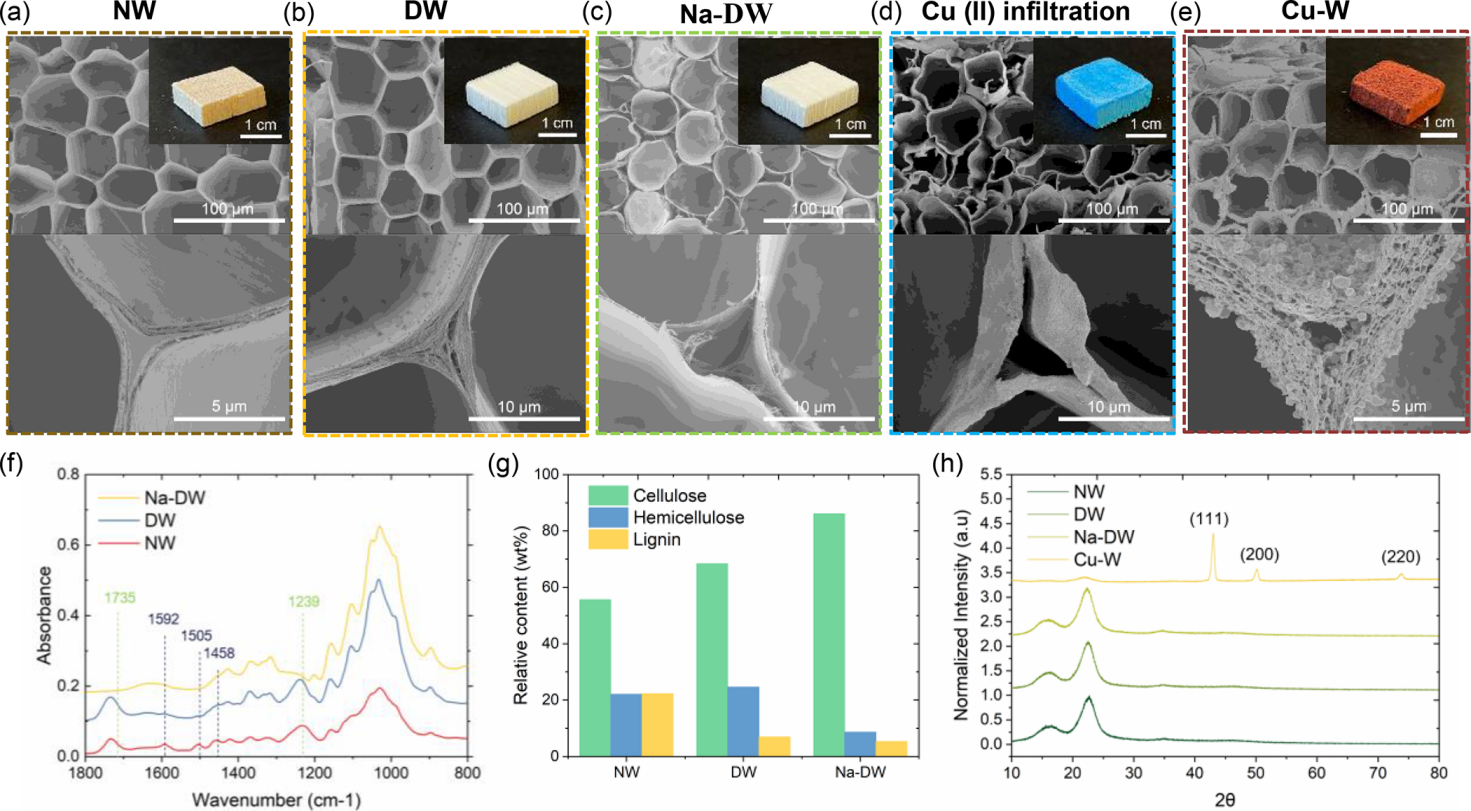
Photographs and SEM images of (a) NW, (b) DW, (c) Na-DW, (d) Cu(II)-infiltrated Na-DW, and (e) Cu-W. (f) FTIR spectra of NW, DW, and Na-DW. (g) Carbohydrate analysis of NW, DW, and Na-DW. (h) XRD patterns of NW, DW, Na-DW, and Cu-W.

Balsa wood was chosen as the starting material because of its high porosity (90%), derived from thin cell walls (0.5–1.5 μm) and large lumen space, as shown in Figure 2a. In the cell wall, cellulose microfibril bundles are embedded in the matrix of hemicellulose and lignin. The middle lamella is a lignin-rich area, and the lignin content gradually decreases from the middle lamella to the secondary wall.^30^ Parts b–e of Figure 2 show the physical appearance and microstructure change of balsa wood during the experimental steps for metallization.

Delignification using NaClO_2_ could selectively degrade lignin through ring-opening oxidative attack while preserving the carbohydrates.^31^ Bleaching effects were observed due to reduced chromophores in the processing.^32^Figure 2b shows that the light-brown color of NW faded away after delignification. Pores were generated in the middle lamellar region and cell wall due to lignin removal. FTIR spectra (Figure 2f) also support the major removal of lignin, as indicated by the absence of characteristic lignin peaks in delignified wood (DW). Specifically, the peaks at 1592 and 1505 cm^–1^ are attributed to aromatic skeletal vibration, and the peak at 1458 cm^–1^ is associated with C–H deformation (methyl and methylene) on lignin. However, there are still small peaks at 1425, 1243, and 1104 cm^–1^, which are related to the C–H in-plane deformation with aromatic ring stretching, C–O of guaiacyl, and C–H of guaiacyl and syringyl, respectively,^33^ demonstrating the minor presence of lignin. Carbohydrate analysis (Figure 2g) also confirms efficient lignin removal. The relative contents of cellulose, hemicellulose, and lignin in NW are 55%, 22%, and 22%, respectively. After delignification, the lignin percentage decreases to 7%, and the cellulose and hemicellulose contents change to 68% and 24% accordingly. XRD was utilized for the crystalline structure of wood (Figure 2h). Four typical crystalline peaks (101, 101̅, 002, and 040) of cellulose I were separated. Figure S1 and Table S2 show the fit peaks upon Gaussian deconvolution and various band positions. The 2θ reflection of 15.2–15.4° is assigned to the (101) crystallographic plane, while 16.7–16.8° is associated with the (101̅) plane. The sharp band of the 2θ reflection at 22.3–22.6° is related to the (002) crystallographic plane of cellulose I.^34^ The broad band at the 2θ reflection of 21.2–21.7° results from amorphous contribution, and a small wide peak at the 2θ reflection of 34.8– 35.1° could be assigned to the (040) crystallographic plane.^35,36^ The CI of DW slightly increased from 56.5% (NW) to 61.7% (Table S2). The major removal of amorphous lignin upon delignification could be an explanation for the increased CI in DW.

Room temperature NaOH treatment was then performed to partially remove hemicellulose for further enhancement of the porosity and accessibility. Hemicellulose is a collection of branched polysaccharides with a low degree of polymerization.^3^ NaOH could partially break the ester bonds between lignin and hemicellulose^37^ and disrupt intermolecular hydrogen bonds between cellulose and hemicellulose.^38^ The major component of hemicellulose in balsa wood is xylan. Most xylose residues contain *O*-acetyl groups, which will be easily cleaved by alkali. The influence of the treatment time on samples (morphology, density, porosity, and SSA) was carried out, as shown in Figure S2. Upon treatment, wood cells lose their connection between each other, leading to a change from the original hexagon or pentagon shape (Figure 2a) to circular cells (Figure S2a–e). The cells separate, and further cell-wall porosity ensues (Figure 2c). Extension of the treatment time made cells a bit more irregular and gave them even further separation (Figure S2a–e). Obvious swelling along the tangential direction was noticed. Compared to DW, the volume of NaOH-treated wood (Na-DW) from 10 min to 1 h expanded around 12.3–14%, while further treatment led to sample shrinkage (Figure S2f). Chemical structure variation upon NaOH treatment was investigated via FTIR (Figure 2f). The band at 1735 cm^–1^, which is associated with carbonyl, is absent, possibly suggesting that the acetyl groups in xylan were removed after NaOH treatment.^38^ The absence of 1230 cm ^–1^ is related to C–O stretching, implying the partial removal of hemicellulose as well.^2^ Carbohydrate analysis (Figure S2) indicated the hemicellulose removal capacity of NaOH. After 10 min of soaking in 7 wt % NaOH, the hemicellulose content decreases from 24.6% to 11.5%, suggesting fast hemicellulose hydrolysis and dissolution in alkali. Extending the treatment time to 1 h further decreased the hemicellulose content to 8.6%. Beyond 1 h, minor hemicellulose removal was observed (8.5% in 2-h-treated samples), which possibly is due to the reactivity and solubility limit of hemicellulose in 7 wt % NaOH. A maximum SSA was obtained for the sample with 1 h of NaOH treatment, which was 13.7 m^2^/g (Figure S2). Taking these factors into consideration, a 1 h treatment will be representative of Na-DW in this work. The CI for Na-DW is 60.9%, no obvious change compared with that of DW (Table S2). In the literature, similar approaches have been carried out for the removal of cell-wall components. However, the reported NaOH treatment was, in general, under high temperature (80–100 °C) for long hours (5–8 h),^21,22^ resulting in a destroyed cell structure. The method here is mild, and the wood-cell-wall structure with multiscale porosity is well preserved.

Metallization was then performed via electroless plating. Na-DW was first soaked in a plating bath without a reducing agent overnight to ensure the thorough infiltration of Cu(II). As shown in the inset of Figure 2d, the white template turns into sapphire blue. Wood cells still have irregular shape and are detached from each other (Figure 2d). The SEM image was captured on a cross section in the middle of the samples, and both sides of the cell walls are covered with tightly packed small crystals, demonstrating complete penetration of the Cu(II)-EDTA complex. EDX mapping on the cell walls also confirms the presence and wide distribution of Cu, as presented in Figure S3.

**Figure 3:**
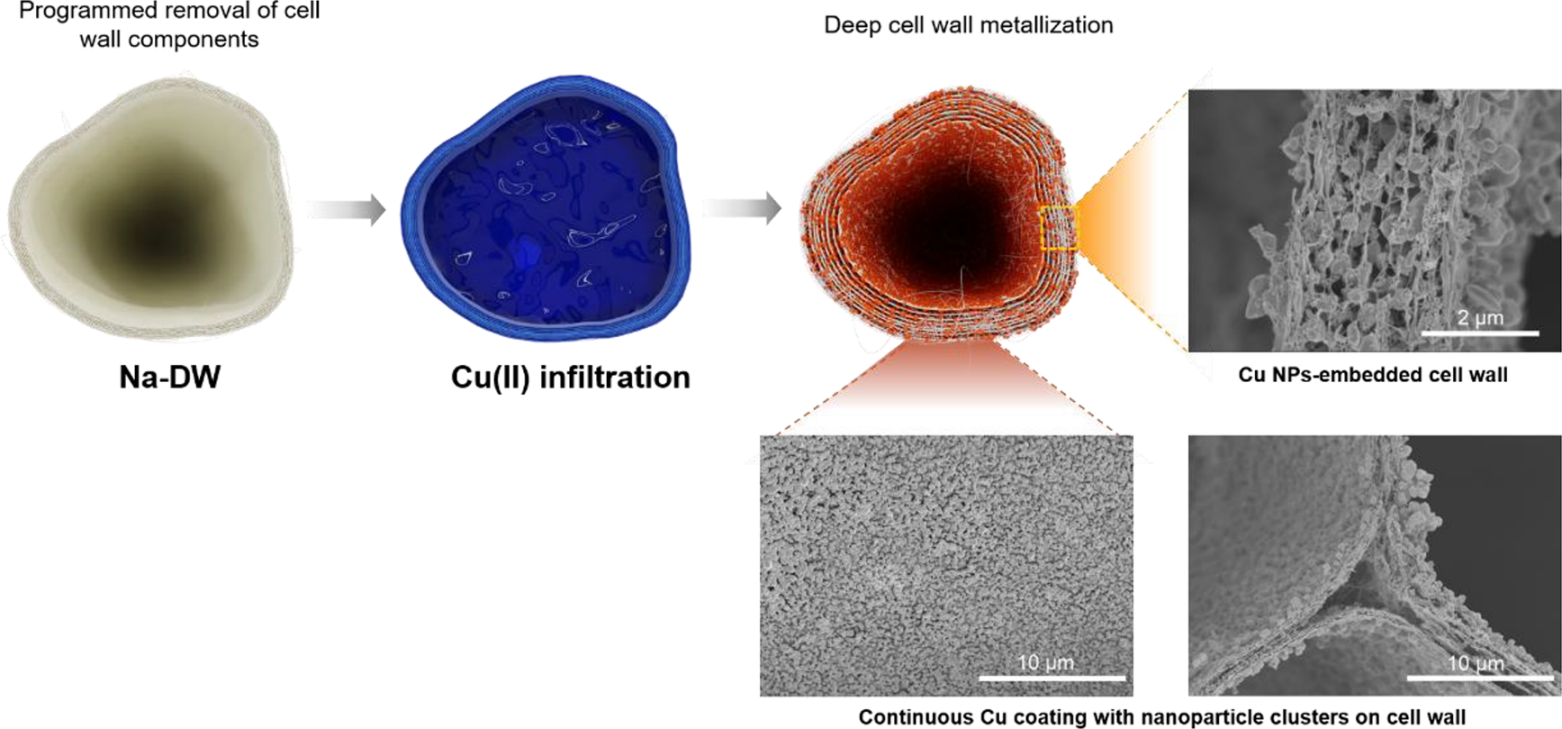
Formation of a deep metallized cell with a multilayered cell wall embedded with Cu NPs. The SEM images on the right side and bottom show that the cell wall was delaminated by NPs and covered by a continuous Cu coating with NP clusters on top of it.

The addition of DMAB induced a series of electrochemical reactions^25^ (Table S1). An optical photograph of Cu-W is inset in Figure 2e, representing a well metal-like coating with red-brownish color. SEM images were captured on the cross section, located in the middle of the Cu-W. Cu NPs and particle clusters were found not only on both sides of the cell wall, even penetrating inside the cell wall. This message indicates the deep metallization of thick wood templates and the effectiveness of programmed cell-wall components removal. It is worth noting that the multilayered cell-wall structure with Cu NPs embedded is generated (Figures 2e and 3), which has rarely been reported. The multiple layered structure most likely results from the original layout of the cell wall. During formation of the S2 layer (thickest secondary cell-wall layer), cellulose microfibrils aggregate together and gain a parallel arrangement in a concentric lamellar pattern.^39,40^ As demonstrated in Figure 3, Cu(II) infiltration resulted in the penetration into and adsorption of Cu(II) onto the lamellar S2 layer. Subsequent reduction led to the in situ synthesis of Cu NPs, facilitating formation of the multilayered structure with Cu NPs insertion, while on the cell wall, the penetration and in situ reduction of Cu(II) caused continuous Cu coating with large Cu NPs clusters on top of it (SEM images at the bottom in Figure 3). Particle–fibril interaction could also be observed, indicating the effect of deep-cell-wall metallization. More SEM images of a continuous Cu coating and a delaminated cell-wall embedded with NPs can be found in Figure S4. The XRD pattern of Cu-W confirms the synthesis of elemental Cu (Figure 2h). The sharp, intensive peaks at 43.3°, 50.4°, and 74.1° correspond to (111), (200), and (220) of the face-centered-cubic structure of Cu, respectively, according to JCPDS 89-2838.^41^ In addition, the characteristic bands of cellulose I are still present.

**Figure 4:**
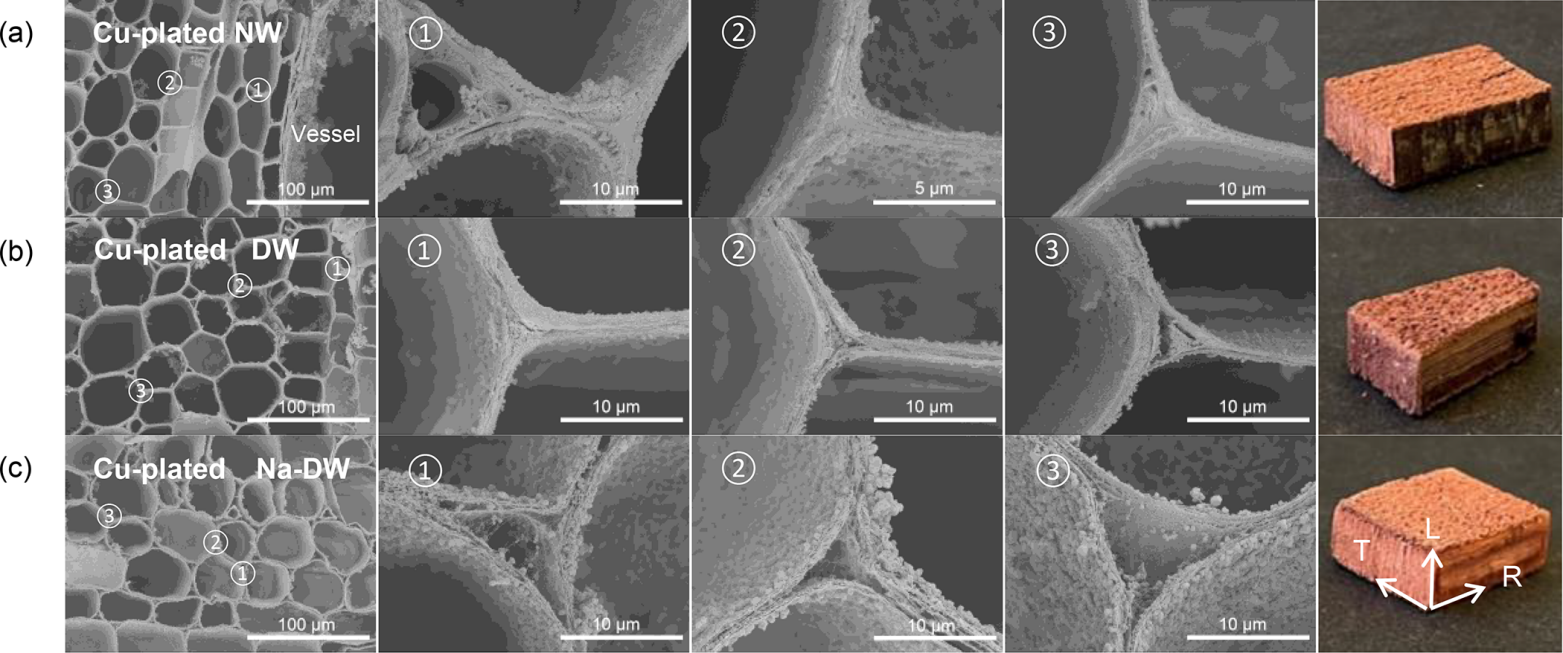
SEM images and section photographs of (a) Cu-plated NW, (b) Cu-plated DW, and (c) Cu-plated Na-DW. L, T, and R stand for the longitudinal, tangential, and radial directions.

The influence of chemical diffusion on wood metallization was further investigated in detail. Figure 4 depicts the morphology of Cu coating on varied templates. For Cu-plated NW (Figure 4a), abundant NPs and clusters are deposited on the cell wall of vessel. The vessels are large (220–330 μm), open-ended cells in hardwood. On the one hand, the dimensions of the vessel and vast number of pits on the vessel cell wall are favorable for chemical diffusion and particle precipitation. On the other hand, the ends of the vessels are wholly or partly open, so are the perforation plates between the vessels.^18^Figure S5 shows the distinct size and structure of the vessels. As a result, chemicals easily go through the vessels, so does the deposition of the particles. Three cell-wall corners were chosen to check the distribution of Cu NPs. For the cell-wall corner close to the vessel (spot 1), many particles still precipitated here. However, for positions a bit farther away from the vessel, deposited particles get fewer and fewer, like spots 2 and 3. A random section of Cu-plated NW, as shown in the photograph in Figure 4a, presents a dark-brown color, which is totally different from the surface, implying a deposition difference and diffusion limit in this sample. Concerning the morphology of Cu-plated DW (Figure 4b), particles accumulated on the cell wall and in the middle lamella. Cell-wall corners were found to have plenty of particle deposition, suggesting the improvement of diffusion. The color of the macroscopic section is closer to that of the surface. For Cu-plated Na-DW (Cu-W), more and larger particle clusters deposited on the cell wall and delaminated the cell wall into multiple layers, as displayed in Figure 4c. Almost all cell walls have similar multilayered microstructures with particles embedded in it. The color variation from the surface and inner section of the sample was negligible, implying the addressing of the diffusion limit in the wood template.

**Figure 5:**
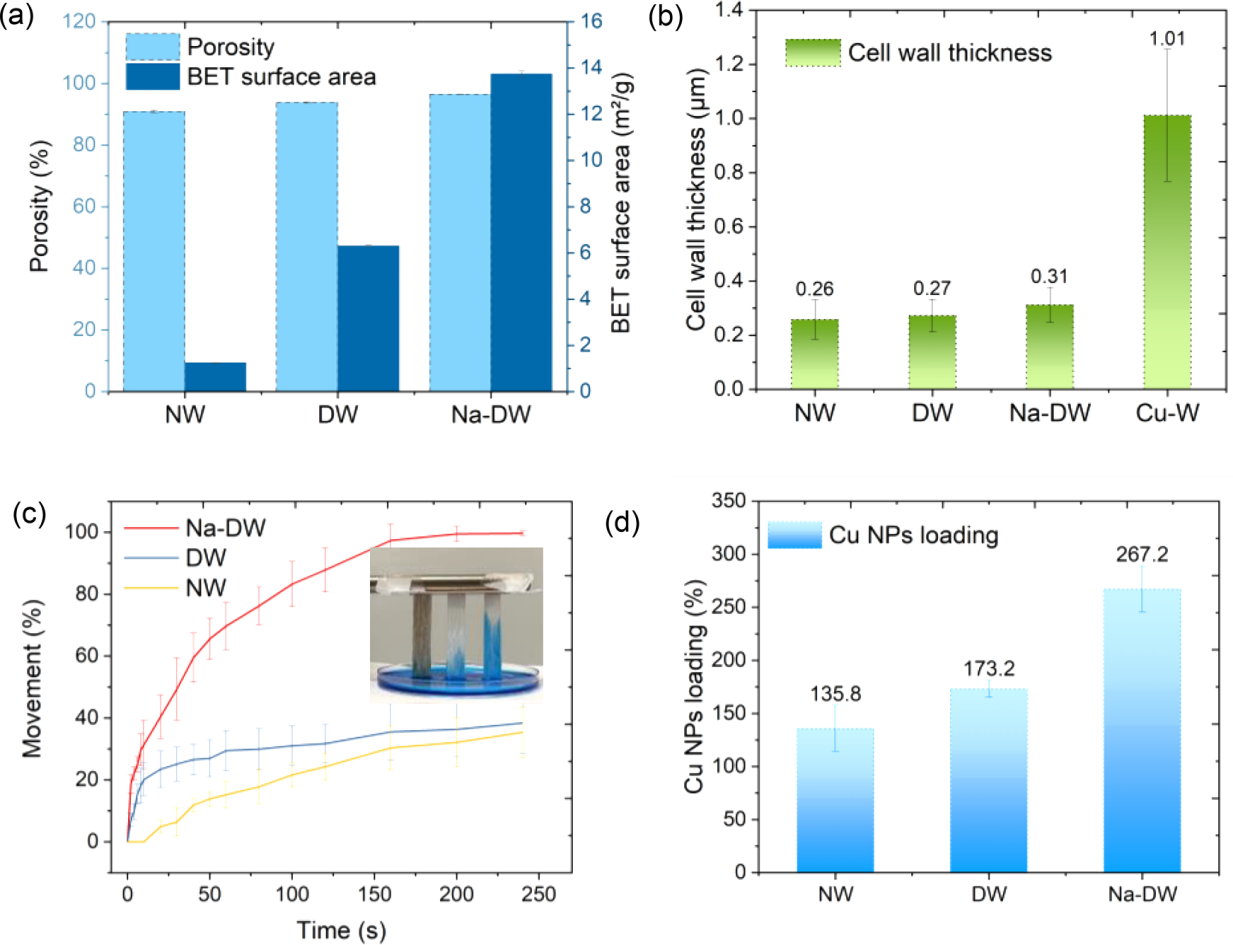
(a) Porosity and SSA of NW, DW, and Na-DW. (b) Cell-wall thickness variation of NW, DW, Na-DW, and Cu–W. (c) Diffusion test on NW, DW, and Na-DW (the inset image was captured when various templates were dipped in a Cu(II) solution for 1 min). (d) Cu NPs loading on Cu-plated NW, DW, and Na-DW.

The achievement of deep-cell-wall metallization is mainly due to the enhanced cell-wall accessibility and chemical diffusion in wood through programmed control of the cell-wall nanostructure. Figure S6 exhibits the absorption and desorption curves of different wood templates. All isotherm plots are type III, indicating that NW, DW, and Na-DW are macroporous materials. Delignification partially removed the cell-wall constituents, making the cell wall more porous. Accordingly, the absorbed N_2_ quantity in DW in BET measurement increased compared to that of NW (Figure S6). The porosity of DW was raised to 94% from 91% (NW), and its SSA also increased to 6.3 m^2^/g from 1.2 m^2^/g (NW) (Figure 5a). NaOH treatment further raised the porosity and SSA of Na-DW to 96.5% and 13.7 m^2^/g, respectively. Additional hemicellulose removal, separated cells (Figure 2c), and swelled cell wall (Figure 5b) could explain the improvement of accessibility.

The enhanced cell-wall accessibility could also be reflected by faster Cu(II) infiltration and higher Cu NPs loading. Figure 5c displays the diffusion rate of Cu(II) through different wood templates, quantified by the ratio of the moving distance of the blue solution. The Cu(II) solution traveled very fast in Na-DW and DW at the beginning, while there was no apparent sign of diffusion in NW until 20 s. The inset photograph in Figure 5c was captured once templates were dipped in a Cu(II) solution for 1 min, at which Cu(II) in Na-DW covered 60–70% of wood template but only 20–25% for DW and 15% for NW. Upon dipping in a Cu(II) solution for 200 s, Cu(II) almost reached the top of Na-DW, whereas the movement ratio was less than 40% for both DW and NW. The complete diffusion process can also be found in the attached video (Video S1). The movement of the Cu(II) solution was driven by capillary force, which could be determined by the pore structure, surface tension of the liquid, and adhesive forces between the liquid and solid surfaces. Although more characterization of the surface chemistry is required, it is reasonable to conclude that programmed removal of the cell-wall constituents is favorable for the diffusion of Cu(II). Figure 5d lists Cu NPs loading on various templates after Cu plating. The particle loading of Na-DW is 267.2%, almost twice that of NW (136%), also suggesting that the accessibility of wood cells has been greatly enhanced.

**Figure 6:**
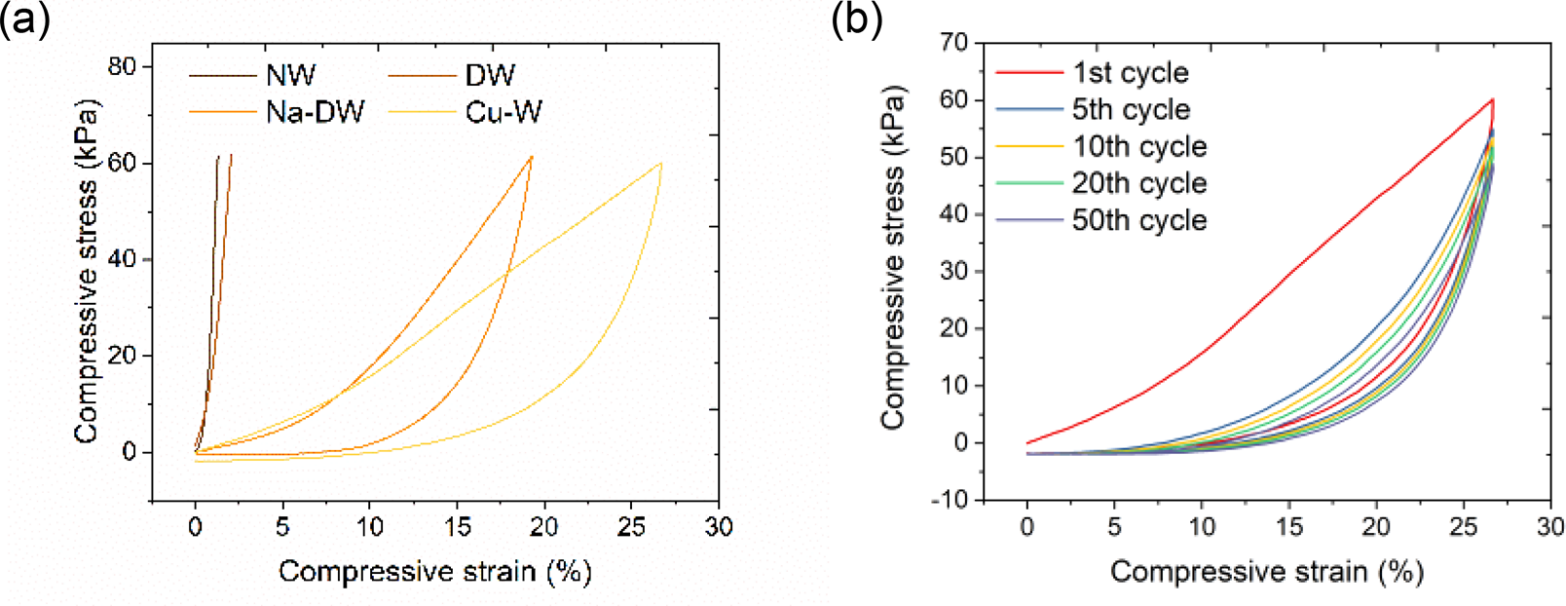
(a) Compression tests on NW, DW, Na-DW, and Cu-W along the tangential direction. (b) Cyclic compression on Cu-W.

The mechanical properties of NW, DW, Na-DW, and Cu-W were evaluated by compression testing along the tangential direction. As shown in Figure 6a, Na-DW and Cu-W possess distinct compressibility. The application of 60 kPa stress led to a strain of 1.3% for NW but 2.0% for DW, demonstrating their rigidity. The slight difference results from decreased integrity due to the partial removal of lignin. For Na-DW, there was a strain of 19.3% upon 60 kPa stress. Partial removal of lignin and hemicellulose caused cells to lose their supportive polygon shape and connection between each other, endowing cells with compressible and recoverable shape. In the cell wall, cellulose aggregates also lost their adhesion between each other, offering more space for compressibility. As a result, programmed removal of the cell-wall constituents endows wood templates with compressible performance. It is worth noting that the compressibility of Na-DW only derives from the tangential direction, showing the anisotropic alignment of cells in wood templates. Cu-W reached an even higher strain of 26.5%, indicating an improvement upon Na-DW and corresponding to a 20-fold increase on compressibility compared to NW. The main reason is that the growth of Cu NPs and particle clusters delaminated the thin cell wall into multiple layers, further breaking the integrity of the cell-wall structure and cellulose aggregates. This is also reflected by the increased cell-wall thickness (Figure 5b) and clear from SEM images of the sample cross section (Figures 2e and 4c). A cyclic compression test was carried out on Cu-W under a strain of 26% (Figure 6b). A linear elastic region below ∼5% and the following densification region where the stress increased sharply with the strain were found in the compressible stress–strain curves. Elastic deformation came from the reshaped and separated cells due to the removal of cell-wall components. Densification was associated with the impediment from the folding and stacking of cells under compression. The strain cannot go to zero when the stress is released, indicating that there is certain plastic deformation during loading. The reason could be the damaged wood cell walls during compression measurement. Around 23% deformation is able to recover after 50 cycles, implying the good compressibility and structural robustness of the Cu-W sample.

**Figure 7:**
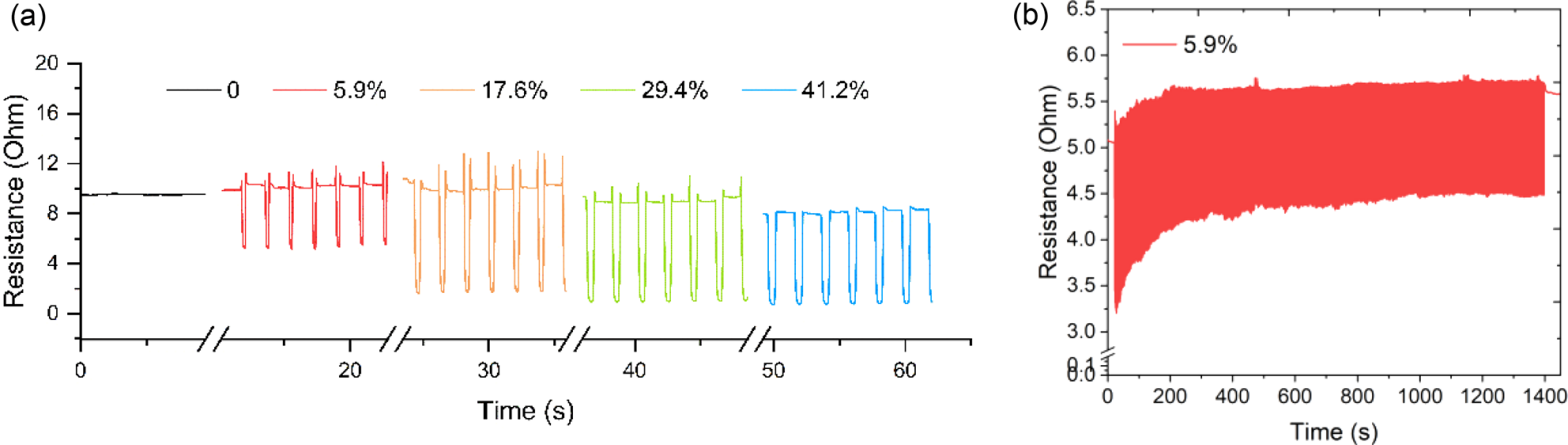
(a) Electrical resistance output of the test sample under various compressive strains. (b) Stability measurement of the test sample as a biobased stress sensor for 1000 cycles.

The compressible Cu-W shows good electrical conductivity as well, making the detection of various electrical signals possible. Cu-W could be mounted on the device with a linear motor (Figure S7a), which would give a defined and periodic pressure on the test sample. Varying strain led to different compressions along the tangential direction, offering diverse electrical resistance. As presented in Figure 7a, the electrical resistance of Cu-W is around 9.5 ohm, and a higher strain resulted in larger compression and more connected channels made of conductive particles and therefore a lower electrical resistance. A strain of 41% could even lower the resistance to 0.8 ohm. The Cu-W-based stress sensor in this study is able to run 1000 cycles under a strain of 5.9%, implying good stability (Figure 7b). The structure stability of Cu-W could be derived from enhanced bonding between the particles and wood substrate. During electroless Cu plating, the diffusion of Cu^2+^ into the cell wall and following in situ reduction generated a great amount of NP–fibril entanglement (Figure S8). Besides, the physical confinement of Cu NPs derived from a multilayered cell wall also contributes to the decent structural stability. A slight increase in resistance was seen at the beginning of the test. The reason could be, on the one hand, that some cells were destroyed by continuous compression and lost part of the conductive channels. In addition, a small amount of Cu particle clusters detached from the wood block because their interaction with the cell wall was broken by applied compression. Further application of the Cu-W sensor for finger movement detection was demonstrated (Figure S7b). The Cu-W sample was connected with a digital multimeter by Cu wires and then attached on a finger. An obvious resistance change was detected during finger tapping or finger bending. Finger movement compressed the cells, causing more conductive Cu particles to contact with each other. As a result, more conductive channels were created for electrons, thus leading to lower resistance. Despite the good performance, there is room to promote metallization in the future for more advanced applications, such as metallization using different metals, enhancing bulk conductivity, etc.

## Conclusions

Wood metallization was explored in this work. Exploiting the structure merits of wood including anisotropy and multiscale porosity, Cu-W was achieved through deep-cell-wall metallization. Effective chemical diffusion secured success by programmed removal of the cell-wall components and tailored reaction kinetics of metallization. Controlled lignin and hemicellulose removal increased the cell-wall porosity and accessibility while lowering the reaction temperature avoided the blockage of pores. As a result, a novel Cu NPs-embedded multilayered cell-wall structure was created in addition to a continuous cell-wall surface coating in the bulk structure. The distinct structure endowed Cu-W with good compressibility and electrical conductivity, making it promising as a stress sensor. A multilayered cell wall embedded with Cu NPs is conducive to the compressibility and stability of the resultant metallic wood. Methodologies reported here could be used for various functionalizations aimed at reaching fine structure in bulk wood, which would open up new possibilities for efficient energy conversion applications and beyond.

## Supporting Information Available

Diffusion testing on NW, DW, and Na-DW (MP4)
Electrochemical reactions during the Cu plating process, Gaussian deconvolution of the XRD pattern for NW, band positions of the crystalline and amorphous cellulose forms of various wood templates, influence of the NaOH treatment time on the morphology, density, porosity, and SSA of specimens, chemical composition and volume expansion of wood templates via different NaOH treatment times, elemental composition of Cu(II)-infiltrated Na-DW, SEM images of a multiple-layered cell wall with Cu NPs embedded, SEM images of vessels, isothermal adsorption–desorption curves of NW, DW, and Na-DW, schematic of a linear motor for measuring variation on the electrical resistance of test samples, demonstration of the resistance variation on Cu-W for finger tapping and bending, and SEM images of particle–fibril entanglement on the cell wall (PDF)

## Abbreviations

Cu-W: metallic Cu wood
DC: direct current
DMAB: (dimethylamino)borane
DW: delignified wood
EDTA: ethylenediaminetetraacetic acid
Na-DW: NaOH-treated wood
NPs: nanoparticles
NW: native wood

## References

[ref1] Chen C.; Zhang Y.; Li Y.; Dai J.; Song J.; Yao Y.; Gong Y.; Kierzewski I.; Xie J.; Hu L. All-wood, low tortuosity, aqueous, biodegradable supercapacitors with ultra-high capacitance. Energy Environ. Sci. 2017, 10 (2), 538–545. 10.1039/C6EE03716J.

[ref2] Sun J.; Guo H.; Ribera J.; Wu C.; Tu K.; Binelli M.; Panzarasa G.; Schwarze F. W. M. R.; Wang Z. L.; Burgert I. Sustainable and Biodegradable Wood Sponge Piezoelectric Nanogenerator for Sensing and Energy Harvesting Applications. ACS Nano 2020, 14 (11), 14665–14674. 10.1021/acsnano.0c05493. 32936611

[ref3] Rowell R. M. Handbook of Wood Chemistry and Wood Composites; CRC Press, 2005. 10.1201/b12487.

[ref4] Wang L.; Li J.; Liu Y. Preparation of electromagnetic shielding wood-metal composite by electroless nickel plating. Journal of Forestry Research 2006, 17 (1), 53–56. 10.1007/s11676-006-0013-5.

[ref5] Liu H.; Li J.; Wang L. Electroless nickel plating on APTHS modified wood veneer for EMI shielding. Appl. Surf. Sci. 2010, 257 (4), 1325–1330. 10.1016/j.apsusc.2010.08.060.

[ref6] Luo W.; Zhang Y.; Xu S.; Dai J.; Hitz E.; Li Y.; Yang C.; Chen C.; Liu B.; Hu L. Encapsulation of Metallic Na in an Electrically Conductive Host with Porous Channels as a Highly Stable Na Metal Anode. Nano Lett. 2017, 17 (6), 3792–3797. 10.1021/acs.nanolett.7b01138. 28463514

[ref7] Huang C.; Grant P. S. Coral-like directional porosity lithium ion battery cathodes by ice templating. Journal of Materials Chemistry A 2018, 6 (30), 14689–14699. 10.1039/C8TA05049J.

[ref8] Zhu M.; Li Y.; Chen F.; Zhu X.; Dai J.; Li Y.; Yang Z.; Yan X.; Song J.; Wang Y.; et al. Plasmonic Wood for High-Efficiency Solar Steam Generation. Adv. Energy Mater. 2018, 8 (4), 1701028 10.1002/aenm.201701028.

[ref9] Wang Z.-L.; Wang M.; Li L.-X.; Bao Y.-P. Permeation behavior of low-melting-point Sn–Bi alloy in the fiber channel of pine wood. Materials & Design 2020, 196, 109068 10.1016/j.matdes.2020.109068.

[ref10] Hui B.; Li J.; Wang L. Preparation of EMI Shielding and Corrosion-Resistant Composite Based on Electroless Ni-Cu-P Coated Wood. Bioresources 2013, 8. 10.15376/biores.8.4.6097-6110.

[ref11] Li J.; Wang Y.; Tian H.; Qi D.; Wang R. Wood Functional Modification Based on Deposition of Nanometer Copper Film by Magnetron Sputtering. Forest Products Journal 2020, 70 (3), 340–349. 10.13073/FPJ-D-19-00054.

[ref12] Mallory G. O.; Hajdu J. B. Electroless Plating: Fundamentals and Applications; William Andrew, 1990.

[ref13] Li J.; Wang L.; Liu H. A new process for preparing conducting wood veneers by electroless nickel plating. Surf. Coat. Technol. 2010, 204 (8), 1200–1205. 10.1016/j.surfcoat.2009.10.032.

[ref14] Wang K.; Dong Y.; Zhang W.; Zhang S.; Li J. Preparation of Stable Superhydrophobic Coatings on Wood Substrate Surfaces via Mussel-Inspired Polydopamine and Electroless Deposition Methods. Polymers 2017, 9 (6), 218 10.3390/polym9060218. 30970897 PMC6432330

[ref15] Duan X.; Liu S.; Huang E.; Shen X.; Wang Z.; Li S.; Jin C. Superhydrophobic and antibacterial wood enabled by polydopamine-assisted decoration of copper nanoparticles. Colloids Surf., A 2020, 602, 125145 10.1016/j.colsurfa.2020.125145.

[ref16] Teng S.; Siegel G.; Prestgard M. C.; Wang W.; Tiwari A. Synthesis and characterization of copper-infiltrated carbonized wood monoliths for supercapacitor electrodes. Electrochim. Acta 2015, 161, 343–350. 10.1016/j.electacta.2015.02.117.

[ref17] Wang T.; Liu G.; Kong J. Preparation of wood-like structured copper with superhydrophobic properties. Sci. Rep. 2016, 5 (1), 18328 10.1038/srep18328. PMC4676000 26656114

[ref18] Borrega M.; Ahvenainen P.; Serimaa R.; Gibson L. Composition and structure of balsa (Ochroma pyramidale) wood. Wood Science and Technology 2015, 49 (2), 403–420. 10.1007/s00226-015-0700-5.

[ref19] Wang Z.; Malti A.; Ouyang L.; Tu D.; Tian W.; Wågberg L.; Hamedi M. M. Copper-Plated Paper for High-Performance Lithium-Ion Batteries. Small 2018, 14 (48), 1803313 10.1002/smll.201803313. 30328292

[ref20] Zhou Y.; Sheng X.; Garemark J.; Josefsson L.; Sun L.; Li Y.; Emmer Å. Enrichment of glycopeptides using environmentally friendly wood materials. Green Chem. 2020, 22 (17), 5666–5676. 10.1039/D0GC01467B.

[ref21] Guan H.; Cheng Z.; Wang X. Highly Compressible Wood Sponges with a Spring-like Lamellar Structure as Effective and Reusable Oil Absorbents. ACS Nano 2018, 12 (10), 10365–10373. 10.1021/acsnano.8b05763. 30272949

[ref22] Song J.; Chen C.; Yang Z.; Kuang Y.; Li T.; Li Y.; Huang H.; Kierzewski I.; Liu B.; He S.; et al. Highly Compressible, Anisotropic Aerogel with Aligned Cellulose Nanofibers. ACS Nano 2018, 12 (1), 140–147. 10.1021/acsnano.7b04246. 29257663

[ref23] Gawande M. B.; Goswami A.; Felpin F.-X.; Asefa T.; Huang X.; Silva R.; Zou X.; Zboril R.; Varma R. S. Cu and Cu-Based Nanoparticles: Synthesis and Applications in Catalysis. Chem. Rev. 2016, 116 (6), 3722–3811. 10.1021/acs.chemrev.5b00482. 26935812

[ref24] Li Y.; Fu Q.; Yu S.; Yan M.; Berglund L. Optically Transparent Wood from a Nanoporous Cellulosic Template: Combining Functional and Structural Performance. Biomacromolecules 2016, 17 (4), 1358–1364. 10.1021/acs.biomac.6b00145. 26942562

[ref25] Zhu P.; Masuda Y.; Koumoto K. Seedless micropatterning of copper by electroless deposition on self-assembled monolayers. J. Mater. Chem. 2004, 14 (6), 976–981. 10.1039/b311061c.

[ref26] Brunauer S.; Emmett P. H.; Teller E. Adsorption of gases in multimolecular layers. Journal of the American chemical society 1938, 60 (2), 309–319. 10.1021/ja01269a023.

[ref27] Teeäär R.; Serimaa R.; Paakkarl T. Crystallinity of cellulose, as determined by CP/MAS NMR and XRD methods. Polym. Bull. 1987, 17 (3), 231–237. 10.1007/BF00285355.

[ref28] Hermans P. H.; Weidinger A. Quantitative X-Ray Investigations on the Crystallinity of Cellulose Fibers. A Background Analysis. J. Appl. Phys. 1948, 19 (5), 491–506. 10.1063/1.1698162.

[ref29] Gibson L. J. Cellular solids. MRS Bull. 2003, 28 (4), 270–274. 10.1557/mrs2003.79.

[ref30] Meier H. General Chemistry of Cell Walls and Distribution of the Chemical Constituents across the Walls. In The Formation of Wood in Forest Trees; Zimmermann M. H., Ed.; Academic Press, 1964; pp 137–151.

[ref31] Sarkanen K. V. The chemistry of delignification in pulp bleaching. Pure Appl. Chem. 1962, 5 (1–2), 219–232. 10.1351/pac196205010219.

[ref32] Bajpai P. Chlorine Dioxide Bleaching. In Environmentally Benign Approaches for Pulp Bleaching, 2nd ed.; Bajpai P., Ed.; Elsevier, 2012; Chapter 6, pp 135–165.

[ref33] Pandey K. K. A study of chemical structure of soft and hardwood and wood polymers by FTIR spectroscopy. J. Appl. Polym. Sci. 1999, 71 (12), 1969–1975. 10.1002/(SICI)1097-4628(19990321)71:12<1969::AID-APP6>3.0.CO;2-D.

[ref34] Wada M.; Okano T. Localization of Iα and Iβ phases in algal cellulose revealed by acid treatments. Cellulose 2001, 8 (3), 183–188. 10.1023/A:1013196220602.

[ref35] Popescu M.-C.; Popescu C.-M.; Lisa G.; Sakata Y. Evaluation of morphological and chemical aspects of different wood species by spectroscopy and thermal methods. J. Mol. Struct. 2011, 988 (1), 65–72. 10.1016/j.molstruc.2010.12.004.

[ref36] Park S.; Baker J. O.; Himmel M. E.; Parilla P. A.; Johnson D. K. Cellulose crystallinity index: measurement techniques and their impact on interpreting cellulase performance. Biotechnology for Biofuels 2010, 3 (1), 10 10.1186/1754-6834-3-10. 20497524 PMC2890632

[ref37] Konnerth H.; Zhang J.; Ma D.; Prechtl M. H. G.; Yan N. Base promoted hydrogenolysis of lignin model compounds and organosolv lignin over metal catalysts in water. Chem. Eng. Sci. 2015, 123, 155–163. 10.1016/j.ces.2014.10.045.

[ref38] Farhat W.; Venditti R.; Quick A.; Taha M.; Mignard N.; Becquart F.; Ayoub A. Hemicellulose extraction and characterization for applications in paper coatings and adhesives. Industrial Crops and Products 2017, 107, 370–377. 10.1016/j.indcrop.2017.05.055.

[ref39] Fahlén J.; Salmén L. On the Lamellar Structure of the Tracheid Cell Wall. Plant Biology 2002, 4 (3), 339–345. 10.1055/s-2002-32341.

[ref40] Ruel K.; Chevalier-Billosta V.; Guillemin F.; Berrio-Sierra J.; Joseleau J.-P. The wood cell wall at the ultrastructural scale-formation and topochemical organization. Maderas. Ciencia y tecnología 2006, 8 (2), 107–116. 10.4067/S0718-221X2006000200004.

[ref41] Ramyadevi J.; Jeyasubramanian K.; Marikani A.; Rajakumar G.; Rahuman A. A. Synthesis and antimicrobial activity of copper nanoparticles. Mater. Lett. 2012, 71, 114–116. 10.1016/j.matlet.2011.12.055.

