# Supplementary material for: Metallic Wood through Deep-Cell-Wall Metallization: Synthesis and Applications
Source: ACS Appl Mater Interfaces. 2024 Apr 18;16(17):22433–42. doi: 10.1021/acsami.4c02779 (PMC11071041; doi:10.1021/acsami.4c02779)
Supplement: Supplementary file 2 — am4c02779_si_002.pdf [file am4c02779_si_002.pdf]

Supporting Information

Metallic Wood through Deep-Cell-Wall Metallization: Synthesis and Applications

Xiaoying Xu [1], *Jonas Garemark* [1], *Farsa Ram* [1], *Zhen Wang* [1], *Yuanyuan Li* [1]*

[1] Wallenberg Wood Science Center, Department of Fiber and Polymer Technology, KTH Royal Institute of Technology, SE-10044 Stockholm, Sweden

*Corresponding author email: yua@kth.se

Table S1. Electrochemical reactions during Cu Plating process

$$(CH_3)_2NHBH_3 + 2H_2O \rightarrow BO_2^- + (CH_3)_2NH + 7H^+ + 6e^- \quad (1)$$

$$CuEDTA^{2-} + 2e^- + H^+ \rightarrow Cu + HEDTA^{3-} \quad (2)$$

$$CuEDTA^{2-} + 2e^- + 2H^+ \rightarrow Cu + H_2EDTA^{2-} \quad (3)$$

$$2CuEDTA^{2-} + 2e^- + H_2O \rightarrow Cu_2O + 2HEDTA^{3-} \quad (4)$$

$$2Cu^{2+} + 2OH^- + 2e^- \rightarrow Cu_2O + H_2O \quad (5)$$

$$Cu^{2+} + 2OH^- \rightarrow CuO + H_2O \quad (6)$$

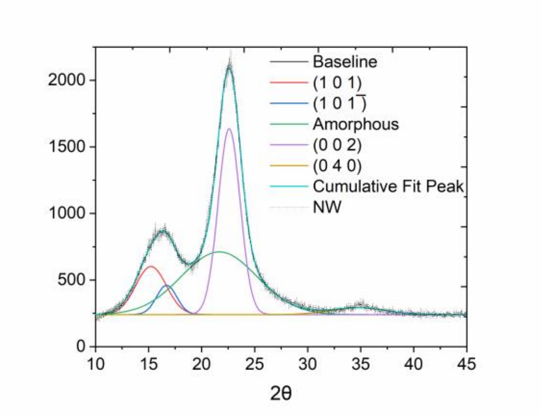

Figure S1. Gaussian deconvolution of XRD pattern for NW

Table S2. The band positions of crystalline and amorphous cellulose forms of various wood templates

Sample	(1 0 1)	$(1\ 0\ \bar{1})$	Amorphous	(0 0 2)	(0 4 0)	CI
NW	15.36	16.85	21.27	22.60	35.07	56.47
DW	15.23	16.78	21.67	22.49	34.84	61.70
Na-DW	15.22	16.85	21.58	22.34	34.80	60.93

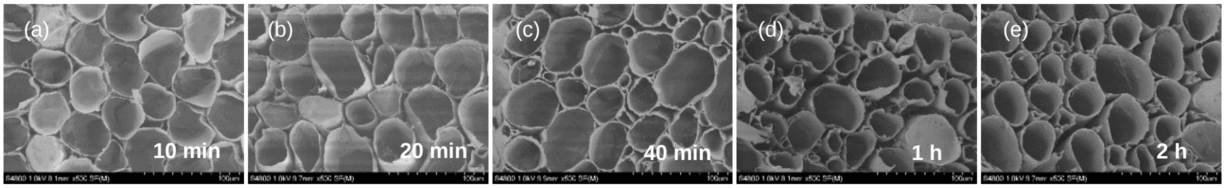

Density: 55.1±0.3 kg/m^3
Porosity: 96.3±0.1%
SSA: 9.6±0.1 m^2/g

Density: 54.9±1.3 kg/m^3
Porosity: 96.3±0.1%
SSA: 10.6±0.1 m^2/g

Density: 53.6±1.8 kg/m^3
Porosity: 96.4±0.1%
SSA: 11.8±0.1 m^2/g

Density: 52.4±1.9 kg/m^3
Porosity: 96.5±0.1%
SSA: 13.7±0.1 m^2/g

Density: 54.7±0.2 kg/m^3
Porosity: 96.4±0.1%
SSA: 8.7±0.2 m^2/g

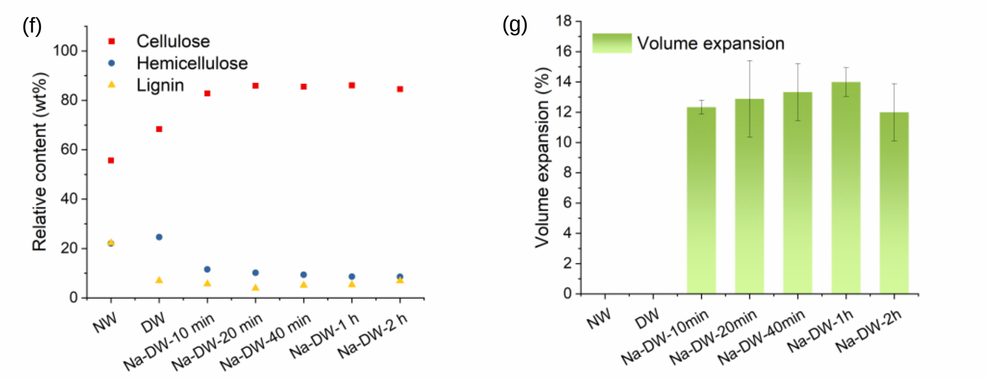

Figure S2. (a)-(e) The influence of NaOH treatment time on the morphology, density, porosity and specific surface area of specimens; (f) chemical composition and (g) volume expansion of wood templates via different NaOH treatment time.

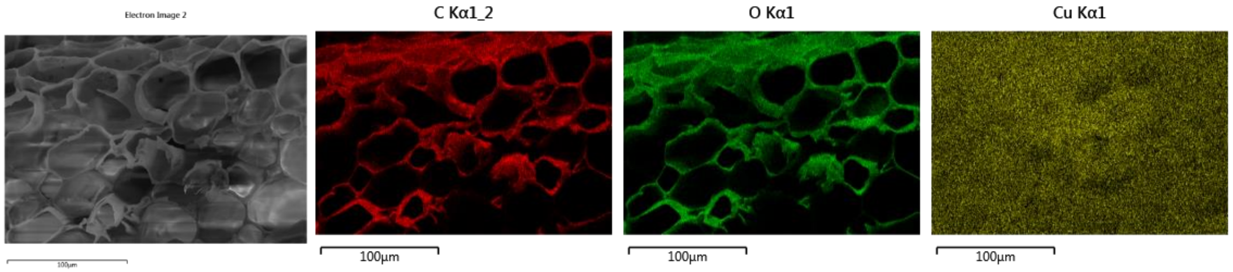

Figure S3. Elemental composition (C, O and Cu) of Cu (II) infiltrated Na-DW

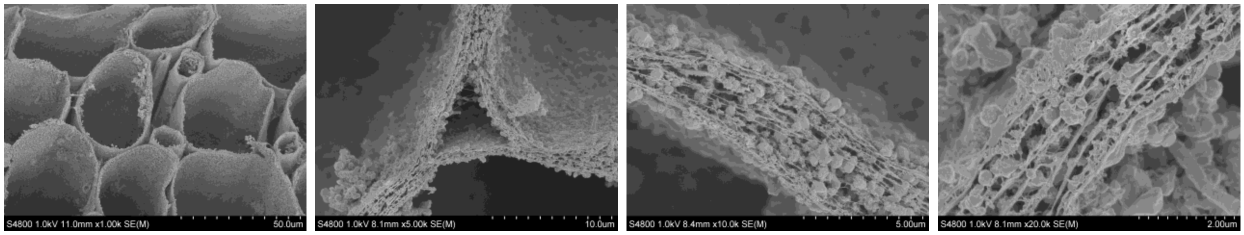

Figure S4. SEM images of multiple-layered cell wall with Cu NPs embedded

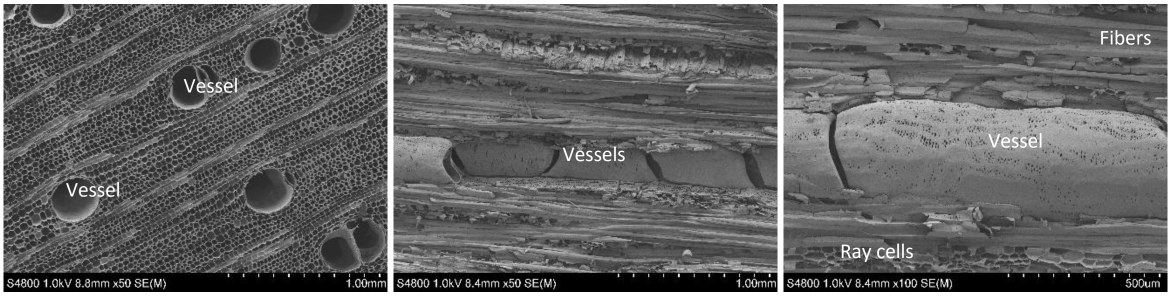

Figure S5. SEM images of vessels with distinct dimensions and structure

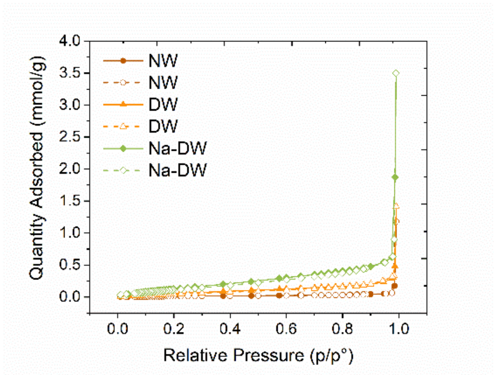

Figure S6. Isothermal adsorption-desorption curves of NW, DW and Na-DW.

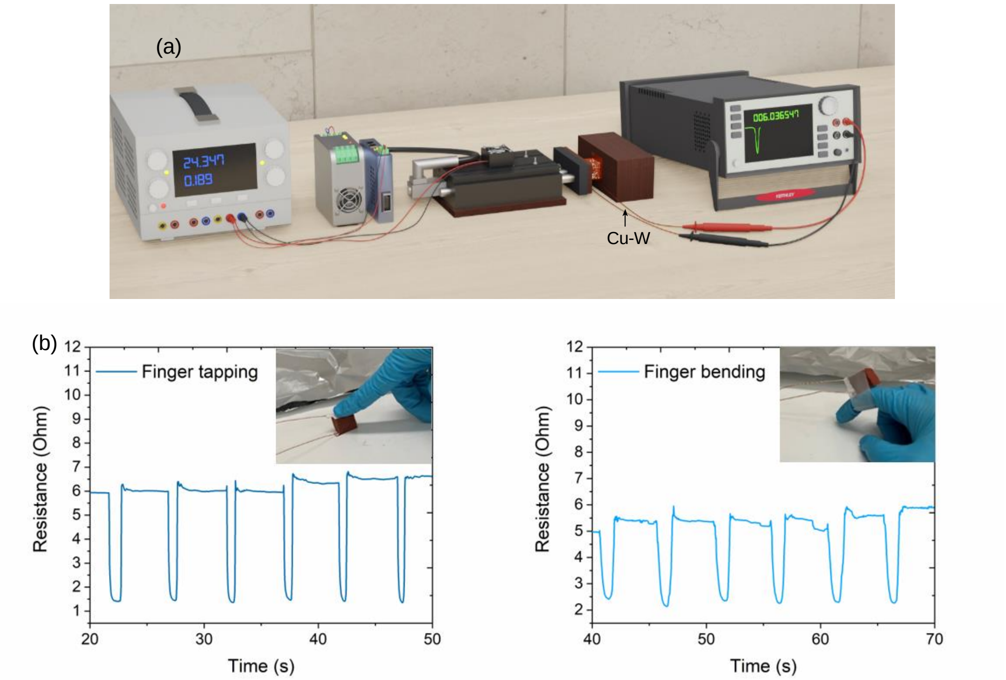

Figure S7. (a) Schematic of linear motor for measuring variation on electrical resistance of test sample. (b) Demonstration of resistance variation on Cu-W for finger tapping and bending

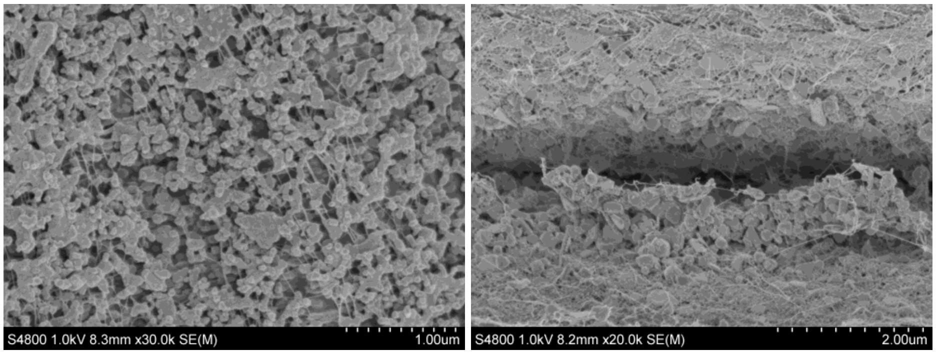

Figure S8. The formed large amount of particle-fibril entanglement on the cell wall during electroless Cu plating.
